# Supplementary material for: Adaptive control for circulating cooling water system using deep reinforcement learning
Source: PLoS One. 2024 Jul 24;19(7):e0307767. doi: 10.1371/journal.pone.0307767 (PMC11268623; doi:10.1371/journal.pone.0307767)
Supplement: Attachment — Submitted filename: Response to Reviewers.docx [file pone.0307767.s002.docx]

Response to Reviewer Comments

Thank you very much for taking the time to review this manuscript. We will carefully consider and provide detailed answers to your questions. Please find the detailed responses below and the corresponding revisions/corrections highlighted/in track changes in the resubmitted files*.*

**Comments 1:** How does the proposed method ensure the stability of the system?

**Response 1**: Thank you for your valuable feedback on our submitted paper. We have carefully read your review comments, and in response to your concerns about system stability, we are willing to provide a detailed response.

In this paper, we employ the Twin Delayed Deep Deterministic Policy Gradient (TD3) algorithm designed to improve training stability. By introducing a Twin Q network and a delayed update mechanism, we aim to reduce the variance during training to prevent excessive fluctuations in the system. The algorithm employs experience playback, which is one of the commonly used techniques in the field of deep reinforcement learning. Experience playback helps mitigate instability due to sample correlation and improves the system's robustness by reusing previous experience. In this paper, we rationally design the state space and the reward function of a multivariate system for circulating cooling water to help the deep learning agent perceive the system state more accurately and adjust it according to the reward signal. This initiative aims to prevent unstable behaviours during the training process. In addition, we introduce a reference trajectory model to accelerate convergence and reduce system oscillations during control. This optimization tool helps to make the control system approximate the optimal policy more smoothly and improve the overall stability.

We encourage regular system performance monitoring in practical applications to enhance safety and system stability further. It is crucial to make adjustments as needed to maintain system stability. Additionally, considering alternative control strategies may be prudent to address specific scenarios that deep reinforcement learning algorithms might not handle effectively. This ensures that the system remains stable even in extreme circumstances.

Based on this, we have made an addition in Section 5. **Please refer to the red content in the first paragraph of section 5 on page 13, at line 311~314.**

**Comments 2:** The authors mentioned many successful applications of RL to circulating cooling water system ([26-28]), what is the contribution of this manuscript compared to them? It is suggested that the motivation and contributions should be more emphasized.

**Response 2:** Thank you for your valuable suggestions on our paper. We understand and value your comments.

Regarding the motivation of the research in this paper, the circulating cooling water system is a complex system with nonlinear, time-lag and multivariate characteristics. Traditional control methods, such as PID controllers, fuzzy control, model predictive control, etc., are often difficult to cope with the complex dynamic characteristics of the system and the uncertainty in the operation process and thus have certain limitations. However, with the development of artificial intelligence technology, reinforcement learning, as a machine learning method based on trial-and-error learning, has powerful nonlinear modelling and adaptive learning capabilities. On the one hand, this paper wants to verify whether deep reinforcement learning has certain advantages over traditional control methods in circulating cooled water systems; on the other hand, although [26-28] have done some research in circulating cooled water-related systems, in general, the research in this field is not deep enough and comprehensive, based on which this paper adopts a different deep reinforcement learning method from [26-28]: the Twin Delayed Deep Deterministic Policy Gradient. The main contributions of this paper are as follows: 1) A deep reinforcement learning controller for circulating cooling water systems was designed based on the TD3 algorithm, achieving end-to-end control and enhancing system stability. 2) The circulating cooling water multivariable system's state space and reward function were reasonably designed. The convergence speed of the agent was accelerated, and the oscillations and instability of the control system were reduced by adding a reference trajectory model. 3) The controller design does not require a model or specialized knowledge about industrial processes. Random disturbance signals were introduced during simulation training to improve the system's adaptive capabilities. 4) The application of deep reinforcement learning in circulating cooling water systems was explored, providing reference and inspiration for control problems in other industrial domains.

Based on this, we have made an addition in Section 1. **Please refer to the red content in the third paragraph of section 1 on page 3, at line 69~83.**

**Comments 3:** Since there are many related methods that can also deal with optimal control of unknown systems, it is better to provide a more comprehensive literature review. Please note that the up-to-date of references will contribute to the up-to-date of your manuscript. The studies named: Robust safe reinforcement learning control of unknown continuous-time nonlinear systems with state constraints and disturbances, Journal of Process Control; Online reinforcement learning with passivity-based stabilizing term for real time overhead crane control without knowledge of the system model, Control Engineering Practice, can be used to explain the method in the study or to indicate the contribution in the "Introduction" section. I believe this would further strengthen the introduction and lend support to the methodology used in general.

**Response 3:** Thank you for your comments and suggestions. The literature you recommended is critical. We fully agree and have added this section to the manuscript.

**Please refer to the red content in the third paragraph of section 1 on page 3, at line 61~68.**

**Comments 4:** Check the notation system throughout the text. For example, the differential operator in equation (1) and the state in MDP use the same character "s". The transfer function G and the state transition function P should be unified, the current expression is confusing. If a1 and M1 represent the same value, why do the authors use different notations?

**Response 4:** Thank you for your comments and suggestions. We apologize for the lack of clarity in our previous presentation; your suggestion is essential.

For this reason, we use "S" to denote the differential operator in Equation (1) and "s" to denote the state in the MDP. The transfer function G is mainly used in control system theory to describe linear time-invariant systems' input and output relationship. In contrast, the state transfer function P is mainly used in the MDP framework to describe the state transfer process between an intelligent body and its environment. In addition, the values of a1 and M1 are indeed the same in this paper, and the reason why different symbols are used is that a1 denotes the value of the action in reinforcement learning, and M1 denotes the value of the valve opening in the circulating cooling water system. The action value a1 obtained after the training of the reinforcement learning algorithm is applied to the circulating cooling water system as a control quantity, and the realization of the control quantity is done through M1.

**For the revision details of this question, please refer to the red content in section 2 on page 5, at line 127.**

**Comments 5:** The control error values in equation (2) are not defined. The error between what? It is suggested that the reference trajectory model be placed in a more appropriate location.

**Response 5:** Thank you for pointing this out. **For the revision details of this question, please refer to the red content in section 3 on page 7, at line 177.**

The reason for placing the reference trajectory model after the setpoint is that, considering that the setpoint may have sudden changes or instability in practical applications, adding the reference trajectory model after it can smooth out the setpoint signal so that its changes are slower and smoother, thus helping to reduce the oscillations and instability of the control system. The fact that the reference trajectory model is placed after the setpoint does not affect the magnitude of the error value.

**Comments 6:** What is the difference between the proposed method and TD3?

**Response 6:** Thank you for pointing this out. We are willing to provide further explanation on the issue.

The control algorithm used in our proposed method is TD3. However, we have adjusted the system's control structure to adapt to the control problems in circulating cooling water systems. Since the setpoints in the circulating water system may have sudden changes or instability in practical applications, this may lead to unstable performance or oscillations in the control system. By introducing a reference trajectory model, the setpoint signal can be smoothed to make its changes slower and smoother, which helps to reduce the oscillation and instability of the control system. From the learning curves of different methods under the same task in Fig. 5, the method proposed in this paper obtains higher rewards faster and more stably due to the addition of the reference trajectory model. Through simulation experiments, the method proposed in this paper has a better performance and a more significant potential in a comprehensive view.

**Comments 7:** Please improve the quality of all figures and the language.

**Response 7:** Thank you for your review and valuable comments. We take your suggestions very seriously and have already started to improve the quality of all graphics and language in the paper. We will carefully review and cross-reference your guidance to ensure that all charts and graphs, as well as the presentation of the paper, are more precise, more accurate, and better aligned with academic requirements. We look forward to demonstrating a tangible effort to improve on your suggested improvements in the final version.

Thank you again for your guidance and review.
