# Supplementary material for: Adaptive control for circulating cooling water system using deep reinforcement learning
Source: PLoS One. 2024 Jul 24;19(7):e0307767. doi: 10.1371/journal.pone.0307767 (PMC11268623; doi:10.1371/journal.pone.0307767)
Supplement: Attachment — Submitted filename: Response to Reviewers.docx [file pone.0307767.s003.docx]

Response to Reviewer Comments

Thank you very much for taking the time to review this manuscript. We will carefully consider and provide detailed answers to your questions. Please find the detailed responses below and the corresponding revisions/corrections highlighted/in track changes in the resubmitted files*.*

**Comments:** Some potential drawbacks of the proposed deep reinforcement learning control method:

1. Complexity: Deep RL methods introduce significant complexity compared to traditional controllers.

2. Hyperparameters: Fine-tuning hyperparameters like discount factor, learning rate etc. requires expertise.

3. Sample efficiency: Large volumes of experience/data needed to learn optimal policy, may not be feasible in practice.

4. Brittleness: Policies could fail under distribution shifts or novel operating conditions not seen during training.

5. Non-stationary systems: No mechanism provided to continually learn as system dynamics change over time.

6. Interpretability: Learned policies are black-boxes, hard to analyze causes of behavior and ensure robustness.

7. Real system validation: Only simulated tests conducted, performance on real plant with noises/disturbances unknown.

8. Computational cost: Training deep RL agents is computationally expensive requiring specialized hardware.

9. Data requirements: Need sufficient coverage of state-action space in collected data to train policy.

10. Safety: No fail-safes described for scenarios where control deteriorates before retraining can occur.

11. Single objective: Only optimize for one control metric, may negatively impact other important factors.

12. Keywords section is missing.

13. Describe dataset features in more details and its total size and size of (train/test) as a table.

14. Flowchart and algorithm steps need to be inserted.

15. Time spent need to be measured in the experimental results.

16. Limitation Section need to be inserted.

17. All metrics need to be calculated in the experimental results as tables.

18. Address the accuracy/improvement percentages in the abstract and in the conclusion sections, as well as the significance of these results.

19. The architecture of the proposed model must be provided

20. The authors need to make a clear proofread to avoid grammatical mistakes and typo errors.

21. The authors need to add recent articles in related work and update them.

22. Add future work in last section (conclusion) (if any)

23. Enhance the clarity of the Figures by improving their resolution.

24. To improve the Related Work and Introduction sections authors are recommended to review this highly related research work paper:

a) Building an Effective and Accurate Associative Classifier Based on Support Vector Machine

b) A survey on improving pattern matching algorithms for biological sequences

**Response**:

Thank you for your detailed comments and suggestions on the paper we submitted. We greatly appreciate your interest and contribution to our work. In your suggestions, we recognize that some of these issues deserve further consideration and improvement.

We greatly appreciate your review and valuable feedback on our work. We acknowledge the challenges you raised **(issues 1-11)** as common concerns faced by deep reinforcement learning methods in the control field. Your insights will help us refine and apply our approach to practical engineering applications. We will carefully consider your suggestions and strive to address these issues in our future work.

Regarding your point about adding a Keywords section **(issue 12)**, we appreciate your suggestion. However, according to the official template and guidelines we followed, a separate Keywords section is optional. For consistency with the format provided by the journal and based on the precedent set by previously published papers on the journal's website, we did not include a Keywords section in our manuscript. Nevertheless, we have provided the relevant keywords for our paper: Industrial process control, Circulating cooling water system, Deep reinforcement learning, PID controller, and TD3.

In response to **issue 13** concerning dataset description, we acknowledge your feedback. Our study did not utilize a specific dataset; instead, we conducted research and experiments based on theoretical models and simulation environments. Therefore, we did not provide specific details about a dataset in the manuscript.

As for your **issue 14**, we are very grateful for your suggestion, which has already been included in the document. Please refer to **Figure 4** and **Algorithm 1**'s related content.

Regarding **issue 15**, we understand your point about time measurement in the experimental results. However, at this stage, we cannot conduct additional time measurements. Therefore, we do not intend to add this information to the manuscript. We will consider incorporating time measurements in future experiments to provide more comprehensive results.

Regarding your **issue 16**, we appreciate your suggestion. Although our approach achieves good control performance in simulation experiments and shows advantages over both traditional control methods and other deep reinforcement learning methods, we are well aware of some potential limitations, such as applicability constraints, computational resource requirements, hyper-parameter sensitivity, adaptability to environmental variations, and the challenges of practical system validation. **Based on this, we have made an addition in Section 5. Please refer to the red content in the second paragraph of section 5 on page 14, at lines 316~320.**

Concerning **issue 17**, we appreciate your recommendation. Performance metrics for controllers, such as rise time, transient time, and overshoot, have been extensively calculated and compared in **Table 2**. The data presented in Table 2 effectively demonstrate the performance disparities among the controllers and offer readers a comprehensive understanding of the experimental outcomes. If you deem additional performance metrics or further analysis necessary, please suggest, and we will make the necessary adjustments.

For **issue 18**, we value your suggestion. The manuscript's abstract, experiments, and conclusion sections thoroughly discuss the significance of our research findings. Thanks again for your advice.

Regarding your **issue 19**, we appreciate your suggestion. Regarding the model of this paper and the system's architecture, which is available in the manuscript, please refer to the relevant part of **Figure 4**.

Concerning your **issue 20 and 23**, we take your suggestions very seriously and have already started to improve the quality of all graphics and language in the paper. We will carefully review and cross-reference your guidance to ensure that all charts and graphs, as well as the presentation of the paper, are more precise, more accurate, and better aligned with academic requirements. Thank you again for your guidance and review.

Regarding your **issue 21**, we appreciate your suggestions. Recent articles on the work studied here are fully cited and discussed in the manuscript, and again, we thank you for your suggestions.

Regarding your **issue 22**, we appreciate and value your suggestion. Regarding the section on future related work, **please refer to the red content in the second paragraph of section 5 on page 14, at lines 320~322.**

Regarding your **issue 24**, thank you very much for reviewing our paper and for the advice you provided. We have carefully considered the references you have given. However, after careful review, they are not directly relevant to the research content of our paper, and therefore, we do not intend to cite them for the time being. We have covered the literature closely related to our research topic in the Related Work and Introduction sections and provided a comprehensive introduction and analysis of related work in the current research area. These references support our paper's background and motivation and give the reader a transparent background and introduction to our research. Please provide specific suggestions if we need to consider citing other relevant literature; we will be more than happy to listen and discuss further.

Thank you again for your valuable suggestions!
