# Supplementary material for: Adaptive control for circulating cooling water system using deep reinforcement learning
Source: PLoS One. 2024 Jul 24;19(7):e0307767. doi: 10.1371/journal.pone.0307767 (PMC11268623; doi:10.1371/journal.pone.0307767)
Supplement: Attachment — Submitted filename: Response to Reviewers.docx [file pone.0307767.s004.docx]

Response to Reviewer Comments

Thank you very much for taking the time to review this manuscript. We will carefully consider and provide detailed answers to your questions. Please find the detailed responses below and the corresponding revisions/corrections highlighted/in track changes in the resubmitted files*.*

**Reviewer #4**

**Comments1:** The methods section provides a detailed description of the proposed approach, including the deep reinforcement learning algorithm, the simulation environment, and the evaluation metrics. However, it would be helpful to provide more details on the implementation of the algorithm, such as the network architecture, the exploration strategy, and the reward function.

**Response1**: Thank you for your valuable comments on our submitted paper. We agree with your suggestions. Please refer to lines 227-237 in Section 3.2 of the manuscript for details on the network architecture of the algorithm. The exploration strategy using Gaussian noise is presented in Table 1. A detailed description of the reward function used in the algorithm can be found in Section 3.1.3.

**Comments2:** It would be useful to provide more information on the simulation environment, such as the size and complexity of the system, and how it was validated.

**Response2**: Thank you for your suggestion. In this study, the system is a two-input, two-output system. We trained the control strategy using the TD3-RTM method and then compared it in detail with traditional PID control, fuzzy PID control, DDPG, and the original TD3 algorithm. To ensure fairness and consistency in the comparison, we set the same initial conditions and disturbance factors. Additionally, we used rise time, settling time, overshoot, and IAE metrics to evaluate the performance of the different control systems.

**Comments3:** It would be helpful to provide all metrics in the experimental results as tables.

**Response3**: Thank you for your suggestion. All metrics in the experimental results (rise time, settling time, overshoot, and IAE) are presented in tabular form. Please refer to Table 2.

**Comments4:** I kindly suggest that you address the accuracy and improvement percentages in the abstract and conclusion sections and highlight the significance of these results. This will provide readers with a clear understanding of the impact of your work.

**Response4**: Thank you for your valuable suggestion. We have revised the abstract and conclusion sections based on your feedback. Please refer to the relevant content in the manuscript.

**Comments5:** It would be useful to provide more details on the implementation of the reinforcement learning algorithm, such as the reward function and the network architecture.

**Response5**: As mentioned in your first question, we provided a detailed description of the reward function used in the algorithm in Section 3.1.3. Please refer to lines 227-237 in Section 3.2 of the manuscript for the algorithm's network architecture.

**Comments6:** To enhance the manuscript's clarity, I recommend that you add more details about the theoretical models and simulation environments in the experiments section. This will enable readers to better understand the methodology behind your research and potentially replicate your experiments.

**Response6**: Thank you for your feedback. All simulation experiments in this paper were conducted on a PC running Windows 11, equipped with an AMD 4600H CPU @ 3.00 GHz and 16GB of RAM, using MATLAB/Simulink R2022b. The design of the control system (Figure 4) and the settings of algorithm-related hyperparameters have been thoroughly explained in the article. We believe this information will enable readers to better understand the methods behind our research and replicate our experiments.

**Reviewer #6**

**Comments1:** In the abstract part, the method adopted by the author is better than the other control strategies, but the author does not specify the control performance index.

**Response1**: Thank you for your valuable feedback on our submitted paper. We have revised the abstract to provide a detailed description of the control performance metrics, as per your suggestion. Please review the updated abstract section in the manuscript.

**Comments2:** There is a syntax error in the introduction, please revise it, between lines 22 and 43.

**Response2**: Thank you for your feedback. The relevant section has been modified accordingly. Please refer to lines 28-50 in the introduction section for the updated content.

**Comments3:** The description between lines 75 and 87 is not appropriate in the introduction, please reconsider.

**Response3**: Thank you for your feedback. The relevant section has been modified accordingly. Please refer to lines 76-92 in the introduction section for the updated content.

**Comments4:** Table 2 should be a three-wire table.

**Response4**: Thank you for your suggestion. Table 2 has been revised.

**Comments5:** The conclusion lacks clarity and should be described objectively.

**Response5**: Thank you for your suggestion. The conclusion section has been revised and objectively described.

**Reviewer #7**

**Comments1:** This article aimed to improve the performance of the circulating cooling system. The motivation of this paper should be based on the application. Why is TD3 suitable for the system?

**Response1**: Thank you for your valuable feedback on our submitted paper. We chose the TD3 algorithm for controlling the circulating cooling water system because it can handle continuous action spaces, utilizes twin Q networks to reduce overestimation bias, and employs delayed policy updates and soft updates to minimize function approximation errors, thereby providing more stable and accurate control. Additionally, TD3's deep neural networks can effectively model the complex nonlinear and multivariate characteristics of the system, enabling real-time adaptation and optimized control, ultimately enhancing system performance. The content has been supplemented in the new manuscript. Please refer to lines 168-174 on page 7 of the manuscript.

**Comments2:** What are the differences between the standard TD3 algorithm, and the proposed algorithm shown in Algorithm 1? The authors did not provide any details about the improvement.

**Response2**: The standard TD3 algorithm is not different from Algorithm 1; this study did not modify the TD3 algorithm itself. Instead, it introduced a reference trajectory model to the circulating cooling water system and designed an adaptive control structure for the Twin Delayed Deep Deterministic Policy Gradient algorithm (TD3-RTM) based on this model, as illustrated in Figure 4. The description of this aspect was not sufficiently clear in the original manuscript, but it has been detailed in the newly submitted manuscript.
